# Twice-daily Budesonide 2-mg Foam Induces Complete Mucosal Healing in Patients with Distal Ulcerative Colitis

**DOI:** 10.1093/ecco-jcc/jjv208

**Published:** 2015-11-16

**Authors:** Makoto Naganuma, Nobuo Aoyama, Yasuo Suzuki, Haruo Nishino, Kiyonori Kobayashi, Fumihito Hirai, Kenji Watanabe, Toshifumi Hibi

**Affiliations:** ^a^Center for Diagnostic and Therapeutic Endoscopy, School of Medicine, Keio University, Tokyo, Japan; ^b^Gastrointestinal Endoscopy and Inflammatory Bowel Disease Center, Aoyama Medical Clinic, Hyogo, Japan; ^c^Department of Internal Medicine, Sakura Medical Center, Toho University, Chiba, Japan; ^d^Colo-Proctology Center, Matsushima Clinic, Kanagawa, Japan; ^e^Department of Research and Development Center for New Medical Frontiers, School of Medicine, Kitasato University, Kanagawa, Japan; ^f^Department of Gastroenterology, Fukuoka University Chikushi Hospital, Fukuoka, Japan; ^g^Department of Gastroenterology, Osaka City General Hospital, Osaka, Japan; ^h^Center for Advanced IBD Research and Treatment, Kitasato Institute Hospital, Kitasato University, Tokyo, Japan

**Keywords:** Ulcerative colitis, budesonide foam, mucosal healing

## Abstract

**Background and Aims:**

Mucosal healing is an important therapeutic goal for ulcerative colitis. Once-daily administration of budesonide 2-mg foam is widely used for inducing clinical remission. No study has assessed the usefulness of twice-daily budesonide 2mg foam on mucosal healing in ulcerative colitis patients. We explored the efficacy for mucosal healing of once- or twice-daily budesonide foam in distal ulcerative colitis patients.

**Methods:**

This study was a multicentre, randomised, double-blind, placebo-controlled trial. In all, 165 patients with active, mild to moderate distal ulcerative colitis were randomised to three groups: once- or twice-daily budesonide 2mg/25ml foam, or placebo foam, for 6 weeks. Complete mucosal healing [endoscopic subscore = 0] and the safety profile were assessed at Week 6. Prespecified and post hoc analyses were used.

**Results:**

The percentages of complete mucosal healing in the twice-daily budesonide foam group were 46.4% compared with 23.6% in the once-daily group [*p* = 0.0097], or 5.6% in the placebo group [*p* < 0.0001]. The percentages of clinical remission and the percentages of endoscopic subscore ≤ 1 in the twice-daily budesonide foam group were 48.2% and 76.8%, compared with 50.9% and 69.1% in the once-daily group [no difference], or 20.4% and 46.3% in the placebo group [*p* = 0.0029 and *p* = 0.0007], respectively. In the subgroup of patients with previous use of a 5-aminosalicylic acid suppository or enema, there was a greater percentage of complete mucosal healing in the twice-daily budesonide foam group [32.0%] compared with that in the once-daily [8.7%, *p* = 0.0774] or placebo groups [4.8%, *p* = 0.0763], though there was no significant difference. No serious adverse event occurred.

**Conclusions:**

A significantly greater percentage of patients receiving twice-daily administration of budesonide foam compared with once-daily administration/placebo achieved complete mucosal healing. This is the first study to evaluate the endoscopic efficacy of twice-daily administration of 6-week budesonide foam treatment for ulcerative colitis.

## 1. Introduction

Ulcerative colitis [UC] is a chronic inflammatory bowel disease characterised by recurring episodes of inflammation primarily involving the mucosal layer and occasionally the submucosa of the colon. UC is characterised by clinical symptoms such as rectal bleeding, persistent bloody diarrhoea, and abdominal cramping. The effectiveness of UC therapies is assessed by improvement of these clinical symptoms and mucosal inflammation. Recently, among these therapeutic endpoints, achieving mucosal healing has been recognised as the most important therapeutic goal for UC. Mucosal healing leads to sustained clinical remission in the long term, and it decreases the need for corticosteroids, hospitalisation rates, risk of colorectal cancer, and risk of colectomy.^1,2^ Although there is no validated definition of mucosal healing, an international organisation of inflammatory bowel disease task forces defined mucosal healing as the absence of friability, blood, erosions, or ulcers in the colonic mucosa.^1^ However, mucosal healing is often defined as a Mayo endoscopic subscore ≤ 1, which includes erythema and mild friability in recent clinical studies.^3^ Because erythema and mild friability indicate an inflammatory condition, the Mayo endoscopic subscore ≤ 1 does not always mean an inactive condition of the colonic mucosa that should be called healing. Actually, some reports have suggested that the relapse rate of patients who achieved complete mucosal healing [defined as a Mayo endoscopic subscore of 0] was lower than that of patients who achieved only mucosal healing [defined as a Mayo endoscopic subscore of 1].^3,4,5^ Thus, the desired therapeutic goal in UC is complete mucosal healing [defined as a Mayo endoscopic subscore of 0] rather than mucosal healing [defined as a Mayo endoscopic subscore ≤ 1]. However, potent therapeutic options that induce complete mucosal healing remain to be established.

Budesonide foam is a rectal preparation that has been used since 2006 in Europe [2-mg/dose of rectal foam; Budenofalk^®^] and it was newly approved in the USA in October 2014 [2mg of rectal foam; Uceris^®^]. Budesonide is a high-potency corticosteroid with a low systemic effect compared with classical corticosteroids. Because of its safety profile, budesonide agents are preferred used as early treatment for UC and Crohn’s disease rather than classic corticosteroids such as prednisolone.^6,7,8^ The recommended dosage of budesonide 2-mg foam for clinical remission has been determined as once-daily [QD] administration in Europe or twice-daily [BID] administration for 2 weeks followed by QD administration for 4 weeks in the USA. However, its optimal dose for complete mucosal healing remains to be determined.

In this study, we performed a Phase 2 trial to explore the efficacy and safety of once- and twice-daily budesonide foam treatment in Japanese patients and to determine its optimal dose to induce complete mucosal healing.

## Materials and Methods

### 2.1. Study design

The present trial consisted of run-in and treatment phases. The run-in phase was a single-blind phase for a week before the treatment phase started. The run-in phase aimed to reduce the placebo effect following the double-blind treatment phase. Patients whose symptoms improved during the run-in phase were excluded from the treatment phase. The treatment phase was a double-blind study period for 6 weeks starting with randomisation in a 1:1:1 ratio to three groups administered with QD or BID budesonide foam, or placebo foam. Randomisation was performed using a block size of six with a randomisation programme. Patients were dynamically allocated with the minimisation method to each group based on the following randomisation factors: the total stool frequency subscore; rectal bleeding subscore; endoscopic subscore at baseline [3–4 or 5–6]; duration of induction therapy in the present active phase [< 4 weeks or ≥4 weeks]; and extent of the lesion [proctitis or sigmoiditis]. BID administration was performed using placebo foam in the placebo group and budesonide foam [2mg/25 ml] in the BID group. In the QD group, placebo foam was administered in the morning, and budesonide foam [2mg/25 ml] was administered in the evening. All patients, investigators, and funders were blinded until all observations, evaluations, and data collection were completed, and the prespecified statistical analysis plans were finalised.

### 2.2. Patients

This multicentre, randomised, double-blind, placebo-controlled trial was conducted at 40 centres in Japan between September 2012 and December 2013. The clinical trial was conducted according to good clinical practice. The protocol was approved by the institutional review board for each centre. All patients provided written informed consent. Eligible patients were aged 16–69 years with active, mild to moderate UC. The disease activity for each patient was assessed using a Modified Mayo Disease Activity Index [MMDAI] score. A modification was made to the original Mayo Index—friability was deleted from the definition of an endoscopic subscore of 1.^9^ The enrolment criteria were as follows: an endoscopic subscore of 2; rectal bleeding subscore of 1 or 2; stool frequency subscore of 0–2; lesions restricted to the segment from the rectum to the sigmoid colon; and 12 weeks or longer since the confirmed diagnosis of UC. Oral 5-aminosalicylic acid [5-ASA] agents, or oral salazosulfapyridine agents, or probiotics were permitted at stable doses as concomitant therapies. The use of the following drugs and therapies was prohibited during this study: rectal preparations or suppositories of 5-ASA; suppositories of salazosulfapyridine; corticosteroid preparations; cytapheresis; immunomodulators; anti-tumour necrosis factor antibody preparations; and surgical treatment for UC. Patients were excluded from enrolment in this study if they had any of the following: a history of colon resection; irritable bowel syndrome; intolerance or allergic reaction to budesonide; or a plasma cortisol level < 4.0 μg/dl.

### 2.3. Efficacy evaluations

Patients were evaluated at Weeks 0 [baseline], 2, 4, and 6, or at the withdrawal visit. The MMDAI score was assessed at Weeks 0 and 6 by colonoscopy and symptom-recording diary data based on the 3 days closest to each visit. Endoscopic examination was performed by total colonoscopy at Week 0 and total colonoscopy or sigmoidoscopy at Week 6. The endoscopic subscoring by the attending investigator was applied to the analysis data. In addition, the endoscopic subscores were also evaluated by the central committee for endoscopic evaluation, to assure the reliability of the evaluation by the investigators. All evaluations were blindly performed. Complete mucosal healing was defined as an endoscopic subscore of 0. Clinical remission was defined as a rectal bleeding subscore of 0 and endoscopic subscore ≤ 1, and a stool frequency subscore of 0 or a decrease of ≥ 1 in the stool frequency subscore from baseline.

### 2.4. Safety and acceptance evaluations

At each visit, the vital signs were measured, adverse effects were assessed, concomitant therapy was reviewed, and a general laboratory test was performed. Morning plasma cortisol level tests were performed at baseline and Week 6. If adverse events did not resolve by Week 6 or the withdrawal visit, a follow-up survey was administered until the adverse events disappeared. To assess patients’ acceptance at Week 6 or the withdrawal visit, all patients were asked to answer questionnaires about general problems related to the treatment. If patients had experience of using any other enemas, they were requested to answer another questionnaire to compare the other enemas.

### 2.5. Sample size

We referred to the results of a late phase 2 study of budesonide rectal foam in European UC patients [unpublished data]. The estimated percentage of clinical remission in the twice-daily budesonide foam group was 38.7%. With an estimated drop-out rate of 10%, we aimed to include 55 patients in each group and 165 patients in total. It was possible to calculate the clinical remission rate with an accuracy of ± 13.5%. The target sample size was considered acceptable for the objective of exploring the efficacy in each group.

### 2.6. Statistical methods

The demographics and baseline characteristics of patients in this trial were summarised to assess the balance of the three treatment groups by descriptive statistics. The efficacy analysis was performed in the full analysis set consisting of all patients who were enrolled, randomised, received at least one dose of study treatment, and had at least one available efficacy datum. The safety analysis was performed in the safety analysis set consisting of all patients who were enrolled, randomised, and received at least one dose of study treatment.

Efficacy analysis was performed using the logistic regression model, after adjusting for the sum of three MMDAI subscores consisting of: the endoscopic subscore, rectal bleeding subscore, and stool frequency subscore [3–4 or 5–7] at baseline; extent of the lesion [proctitis or sigmoiditis]; and period of present induction therapy [≤ 27 days or ≥ 28 days] to compare the clinical response among treatment groups. To evaluate the percentage of complete mucosal healing, the Cochran-Mantel-Haenszel test, a comparison between the BID and QD groups, and subgroup analysis were performed in post hoc analyses. All statistical tests were conducted at a two-tailed level of significance of 0.05. No adjustment was made for multiple comparisons because the analysis should be considered exploratory.

## 3. Results

### 3.1. Patient disposition, baseline demographics, and clinical characteristics

**Figure 1.**
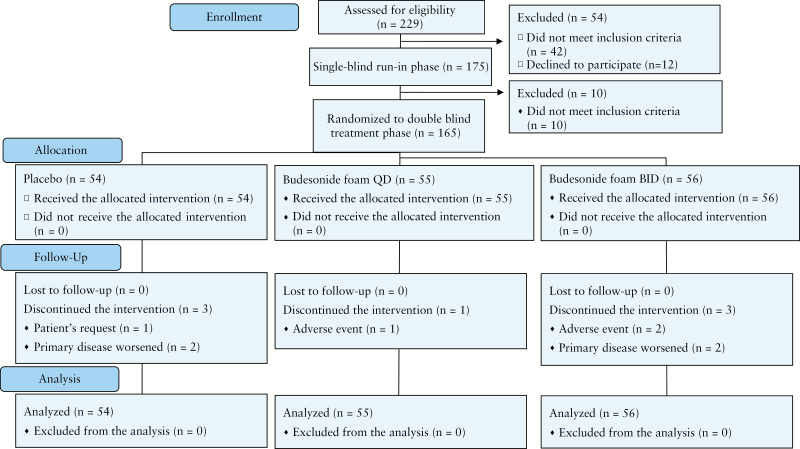
Patient flow diagram. BID, twice a day; QD, once daily.

The disposition of patients is shown in Figure 1. Of 229 patients who gave informed consent, 54 were not enrolled in the single-blind run-in phase. Among the remaining 175 patients, 165 were randomised to the double-blind treatment phase; 158 patients completed the study treatments. All 165 patients were included in the full analysis set for efficacy and safety assessment. Baseline demographics and clinical characteristics were generally balanced among the three randomised groups [Table 1].

**Table 1.** Baseline demographics and clinical characteristics

	Placebo [*N* = 54]	Budesonide foam QD [*N* = 55]	Budesonide foam BID [*N* = 56]	Total [*N* = 165]
Age [years], mean [SD]	39.9 [13.0]	44.7 [13.3]	40.8 [11.2]	41.8 [12.6]
Male sex, *n* [%]	31 [57.4]	32 [58.2]	26 [46.4]	89 [53.9]
Body weight [kg], mean [SD]	61.7 [10.3]	60.7 [13.7]	60.1 [11.5]	60.8 [11.9]
Current smoker, *n* [%]	3 [5.6]	1 [1.8]	5 [8.9]	9 [5.5]
Duration of disease [years], mean [SD]	3.5 [3.5]	5.7 [5.6]	5.8 [5.9]	5.0 [5.2]
Clinical course, *n* [%]				
First attack	8 [14.8]	6 [10.9]	9 [16.1]	23 [13.9]
Relapsing/remitting	46 [85.2]	49 [89.1]	47 [83.9]	142 [86.1]
Duration of present active phase [days], mean [SD]	108.9 [202.4]	86.0 [131.3]	95.3 [140.4]	96.6 [160.0]
<4 weeks, *n* [%]	22 [40.7]	27 [49.1]	27 [48.2]	76 [46.1]
≥4 weeks, *n* [%]	32 [59.3]	28 [50.9]	29 [51.8]	89 [53.9]
Extent of lesions, *n* [%]				
Proctitis	25 [46.3]	26 [47.3]	28 [50.0]	79 [47.9]
Sigmoiditis	29 [53.7]	29 [52.7]	28 [50.0]	86 [52.1]
MMDAI, *n* [%]				
Score 3–5	27 [50.0]	26 [47.3]	25 [44.6]	78 [47.3]
Score 6–8	27 [50.0]	29 [52.7]	31 [55.4]	87 [52.7]
Previous medication for UC — *n* [%]				
Oral 5-ASA	47[87.0]	48[87.3]	44[78.6]	139[84.2]
5-ASA enema or suppository	21[38.9]	23[41.8]	25[44.6]	69[41.8]
Concomitant therapy for UC— *n* [%]				
Oral 5-ASA	47 [87.0]	48 [87.3]	43 [76.8]	138 [83.6]
Oral salazosulfapyridine	0 [0.0]	0 [0.0]	1 [1.8]	1 [0.6]
None	7 [13.0]	7 [12.7]	12 [21.4]	26 [15.8]

MMDAI, Modified Mayo Disease Activity Index; BID, twice a day administration; QD, once daily administration; SD, standard deviation, 5-ASA, 5-aminosalicylic acid; UC, ulcerative colitis.

### 3.2. Efficacy

#### 3.2.1. Complete mucosal healing

**Figure 2.**
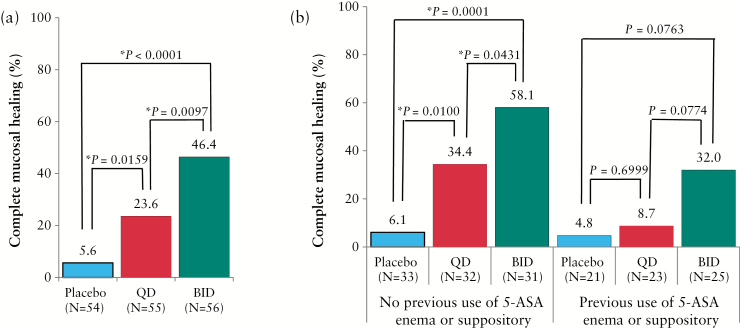
Percentages of complete mucosal healing. [a] Full analysis set. [b] Subgroup analysis of patients with a previous use of 5-ASA enemas or suppositories in the recent active phase. Statistical analyses are performed at a significance level of 0.05 [two-sided]. * Significant difference. BID, twice a day; QD, once daily; 5-ASA, 5-aminosalicylic acid.

The percentages of patients achieving complete mucosal healing in the budesonide foam BID and QD groups were significantly greater than that in the placebo foam group (46.6%, odds ratio [OR] 15.553, *p* < 0.0001 and 23.6%, OR 5.143, *p* = 0.0156, respectively, versus 5.6%). The percentage of patients who achieved complete mucosal healing in the budesonide foam BID group was approximately twice that in the QD group [46.4%, OR 3.024, *p* = 0.0097 versus 23.6%] [Figure 2a]. A significant dose-response relationship for achieving complete mucosal healing was found in the two budesonide foam groups [Cochran-Mantel-Haenszel test, *p* < 0.0001]. The subgroup of patients with previous use of 5-ASA enema or suppository in the recent active phase indicated that these patients were treated by 1-g enemas of 5-ASA or by 1-g suppositories of 5-ASA or by 500-mg suppositories of salazosulfapyridine before enrolment, and entered this study because of insufficient effect of these therapies. Based on the subgroup analysis, the percentages of complete mucosal healing in the budesonide foam BID and QD groups of patients with no previous use of a 5-ASA enema or suppository in the recent active phase were significantly greater than those in the placebo foam group [58.1%, OR 24.867, *p* = 0.0001 and 34.4%, OR 8.354, *p* = 0.0100, respectively, versus 6.1%]. The percentage of patients who achieved complete mucosal healing in the budesonide foam BID group was significantly higher than that in the QD group [58.1%, OR 2.977, *p* = 0.0431 versus 34.4%]. In the subgroup of patients with a previous use of a 5-ASA enema or suppository in the active phase, a greater percentage of complete mucosal healing was observed in the BID budesonide foam group [32.0%] compared with 8.7% in the QD group [OR 4.688, *p* = 0.0774], or 4.8% in the placebo group [OR 7.680, *p* = 0.0763], although a significant difference was not observed [Figure 2b].

### 3.3. Clinical remission

**Figure 3.**
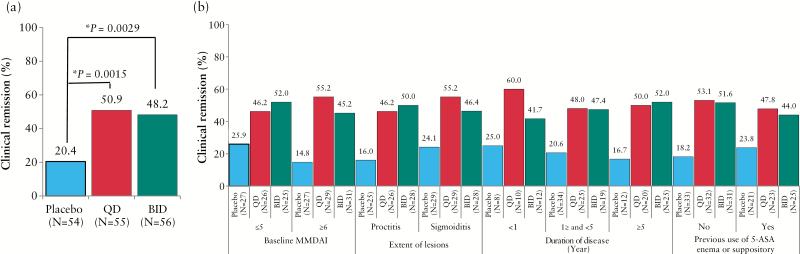
Percentage of clinical remission. [a] Full analysis set. [b] Subgroup analysis. Statistical analyses are performed at a significance level of 0.05 [two-sided]. * Significant difference. BID, twice a day; QD, once daily; 5-ASA, 5-aminosalicylic acid.

At Week 6, the percentages of patients achieving clinical remission in the budesonide foam BID and QD groups were significantly greater than that in the placebo foam group [48.2%, OR 3.674, *p* = 0.0029 and 50.9%, OR 3.994, *p* = 0.0015, respectively, versus 20.4%] [Figure 3a]. The percentages of clinical remission were generally comparable among all subgroups, regardless of the extent of lesions and previous use of a 5-ASA enema or suppository [Figure 3b].

### 3.4. Other endpoints

**Figure 4.**
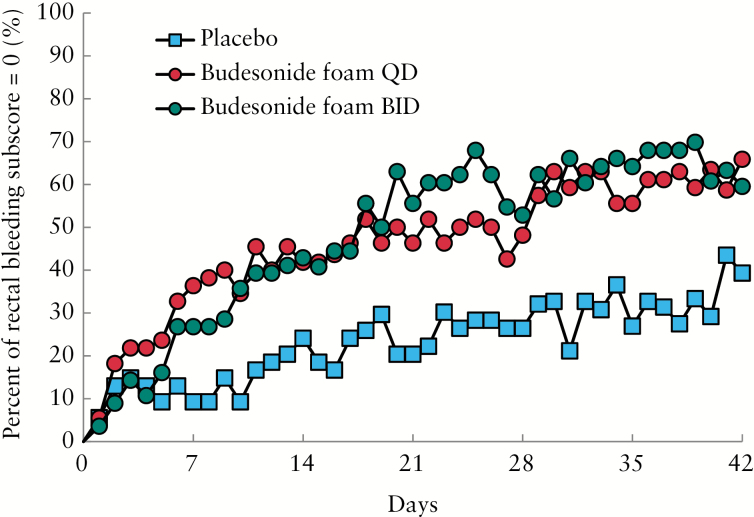
Percentage of patients with a rectal bleeding score of 0 on each day. BID, twice a day; QD, once daily.

The percentages of patients with an endoscopic subscore ≤ 1 in the budesonide foam BID and QD groups were statistically greater than that in the placebo foam group [76.8%, *p* = 0.0007 and 69.1%, *p* = 0.0139, respectively, versus 46.3%] [Table 2]. The percentages of patients with an MMDAI score ≤ 1 in the budesonide foam BID and QD groups were statistically greater than that in the placebo foam group [50.0%, *p* < 0.0001 and 38.2%, *p* = 0.0022, respectively, versus 11.1%]. In both the BID and QD groups, the percentages of elimination of rectal bleeding were higher than that in the placebo group [Table 2, Figure 4].

**Table 2.** Summary of the efficacy endpoints.

	Placebo [*N* = 54]	Budesonide foam QD [*N* = 55]	Budesonide foam BID [*N* = 56]
Endoscopic subscore ≤ 1, *n* [%]	25 [46.3]	38 [69.1]	43 [76.8]
Odds ratio		2.762	4.333
*P*-value		0.0139	0.0007
MMDAI ≤ 1, *n* [%]	6 [11.1]	21 [38.2]	28 [50.0]
Odds ratio		4.966	8.613
*P*-value		0.0022	<0.0001
Rectal bleeding subscore = 0, *n* [%]			
Week 2	13 [24.1]	24 [43.6]	25 [44.6]
Odds ratio		2.497	2.655
*P*-value		0.0307	0.0208
Week 4	15 [28.3]	27 [50.0]	35 [66.0]
Odds ratio		2.552	5.617
*P*-value		0.0276	0.0001
Week 6	19 [37.3]	38 [70.4]	37 [69.8]
Odds ratio		4.240	4.393
*P*-value		0.0010	0.0008

Statistical analyses were performed at the level of significance of 0.05 [two-sided].

MMDAI, Modified Mayo Disease Activity Index; BID, twice a day administration; QD, once daily administration.

### 3.5. Safety

**Figure 5.**
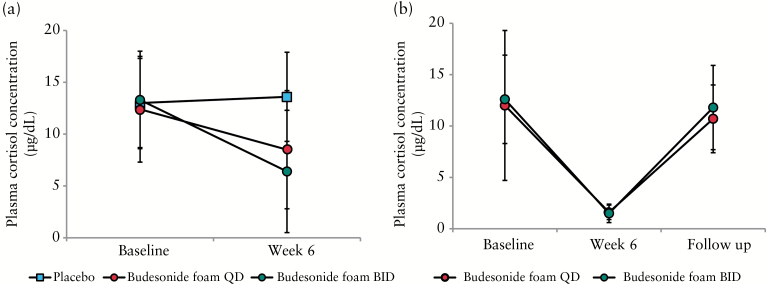
Morning plasma cortisol levels. [a] Morning plasma cortisol levels at baseline and Week 6 in the full analysis set. [b] The recovery of plasma cortisol levels at the follow-up visit. Morning plasma cortisol levels at baseline and Week 6, and the follow-up visit of patients with a decrease of plasma cortisol level at Week 6 [N = 38]. Data presented are mean and standard deviation. BID, twice a day; QD, once daily.

All 165 randomised patients were included in the safety analysis set. A summary of adverse events is shown in Table 3. The incidence of adverse events was 29.6%, 47.3%, and 67.9% in the placebo, QD, and BID groups, respectively, and the incidence of study drug-related adverse events was 11.1%, 30.9%, and 53.6%, respectively. No serious adverse event was reported. The incidence of adverse events leading to treatment discontinuation was 3.7%, 0%, and 3.6% in the placebo, QD, and BID groups, respectively. Regarding symptoms by potential glucocorticoid effects such as a moon face, flushing, fluid retention, mood changes, insomnia etc., there was no evidence of any increase in patients of symptoms caused by glucocorticoids in either the budesonide foam BID or QD group. Among common adverse events, a decrease in the plasma cortisol level and blood corticotrophin level occurred only in the two budesonide foam groups. The incidence of these adverse events was higher in the BID group than in the QD group. The mean plasma cortisol level also decreased in both groups [Figure 5a]. However, in the follow-up survey of 38 patients with a decrease of the plasma cortisol level at Week 6, their plasma cortisol levels returned to the normal level [≥ 4.0 μg/dl] after treatment was terminated [Figure 5b]. Similar results were shown in the follow-up survey of patients with decreases of their blood corticotrophin levels [data not shown].

**Table 3.** Adverse events.

	Placebo [*N* = 54]	Budesonide foam QD [*N* = 55]	Budesonide foam BID [*N* = 56]
Summary of adverse events, *n* [%]			
Adverse events	16 [29.6]	26 [47.3]	38 [67.9]
Study drug-related adverse events	6 [11.1]	17 [30.9]	30 [53.6]
Death	0 [0.0]	0 [0.0]	0 [0.0]
Serious adverse events	0 [0.0]	0 [0.0]	0 [0.0]
Adverse events leading to treatment discontinuation	2 [3.7]	0 [0.0]	2 [3.6]
Common adverse events^a^, *n* [%]			
Infections and infestations	-	-	-
Nasopharyngitis	3 [5.6]	6 [10.9]	4 [7.1]
Nervous system disorders	-	-	-
Headache	1 [1.9]	-	6 [10.7]
Gastrointestinal disorders	-	-	-
Vomiting	-	-	2 [3.6]
Colitis ulcerative	2 [3.7]	-	1 [1.8]
General disorders and administration site conditions	-	-	-
Pyrexia	2 [3.7]	2 [3.6]	-
Investigations	-	-	-
Plasma cortisol decrease	-	12 [21.8]	26 [46.4]
Plasma corticotrophin decrease	-	8 [14.5]	16 [28.6]
Blood creatine phosphokinase increase	-	2 [3.6]	-

^a^ Defined as an adverse event that occurred in at least two patients in any group.

BID, twice a day administration; QD, once daily administration.

### 3.6. Patients’ acceptance

Responses to the questionnaires were collected from 164 of 165 patients who were treated with the study drug. The results of the questionnaires are shown in Table 4; 95.6% of patients said that handling the device was very easy to usual. The majority of patients answered none to slight about having an unpleasant feeling, retention problem, or flatulence [82.9%, 95.1%, and 83.6%, respectively]. Regarding the difficulty in the BID administration, 83.0% of patients answered none to moderate. Of 164 patients, 125 who had experience with other enema preparations answered the same questions from the viewpoint of comparing the foam preparations in this study with other liquid enema preparations. In these patients, a higher percentage of patients answered very easy regarding handling the device [56.8%] compared with all patients [34.1%]. Furthermore, the percentage of patients in the subgroup [55.2%] with experience with other enema preparations, who answered none about having an unpleasant feeling, was higher than that in all patients [28.0%].

**Table 4.** Patients’ acceptance.

	All patients [*N* = 164]	Subgroup with experience of other enemas [*N* = 125]
Handling of device, %		
Very easy	34.1	56.8
Easy	46.3	27.2
Usual	15.2	10.4
Difficult	4.3	4
Very difficult	0	0.8
No answer	0	0.8
Unpleasant feeling, %		
None	28	55.2
Slight	54.9	37.6
Moderate	13.4	3.2
Severe	3	0.8
Very severe	0.6	0.8
No answer	0	2.4
Retention problem, %		
None	75	82.4
Slight	20.1	10.4
Moderate	3	3.2
Severe	1.2	0.8
Very severe	0.6	0
No answer	0	3.2
Flatulence, %		
None	47.6	67.2
Slight	36	24
Moderate	12.2	4.8
Severe	4.3	1.6
Very severe	0	0
No answer	0	2.4
Difficulty of twice daily administration, %		
None	34.8	41.6
Slight	30.5	24
Moderate	17.7	16.8
Severe	14.6	12.8
Very severe	2.4	2.4
No answer	0	2.4

## 4. Discussion

Previous clinical studies have shown that budesonide foam is effective and well tolerated in the treatment of patients with active, mild to moderate UC.^10,11^ However, a few studies have focused on BID administration for 6 weeks or the therapeutic potential to induce complete mucosal healing, because budesonide foam has been commonly used as QD administration in Europe and BID administration for only 2 weeks in the USA. Our study was the first trial to evaluate the efficacy and safety of BID budesonide foam for 6 weeks in terms of achieving complete mucosal healing. A recent study indicated that 55.8% of patients receiving budesonide foam BID for 2 weeks and then QD for 4 weeks achieved an endoscopic subscore of 0 or 1, whereas 76.8% of patients with an endoscopic subscore ≤ 1 in the budesonide foam BID group and 46.4% of patients receiving budesonide foam BID for 6 weeks achieved complete mucosal healing [an endoscopic subscore of 0] in our study. Thus, budesonide foam BID for 6 weeks maybe useful for achieving complete mucosal healing in patients with UC.

In previous studies, it has been shown that there was no significant difference in the clinical remission rates between QD and BID administration of a 2-mg budesonide enema.^12^ On the basis of this study, the recommended dose of a budesonide enema or budesonide foam was QD administration.^10,12^ Meanwhile, a previous dose-ranging study suggested that a > 2-mg dosing of budesonide enema was possibly more effective in sigmoidoscopic inflammation than a 2-mg dosing.^13^ Consistent with these reports, our study suggested that BID administration of budesonide 2-mg foam [4mg/day in total] showed the potential benefit compared with QD administration [2mg/day] in terms of improving colonic inflammation. This difference was not observed when we focused on clinical remission only. On the other hand, in an uncontrolled open-label trial, a total of 12 patients with mild to moderate active distal UC applied a single rectal dose of 99mTc-labelled budesonide 2-mg foam for gamma scintigraphical examination to determine extent of foam distribution within the colon.^14^ The scintigraphy data from this study demonstrated that the greater part of drug cleared in rectum within only 6h, suggesting that twice-daily administration could extend the exposure time of budesonide to the rectum. Thus, the better effect of BID administration of budesonide 2-mg foam was considered not only due to the higher dose of budesonide but also to the twice daily administration.

Our study showed that BID administration of budesonide foam induced complete mucosal healing in approximately half of UC patients. A previous clinical trial in mild to moderate UC patients has reported the percentages of patients with complete mucosal healing after treatment by oral 5-ASA preparations. After treatment for 8 weeks, 32.0% of patients treated with 2.4g of oral 5-ASA and 32.3% of patients treated with 4.8g of oral 5-ASA achieved complete mucosal healing.^9^ In a randomised clinical trial of 5-ASA suppositories, the percentages of patients with complete mucosal healing were 29.0% in QD 5-ASA suppository therapy for 4 weeks.^15^ However, in our study, 46.4% of patients achieved complete mucosal healing after BID budesonide foam therapy for 6 weeks. Whereas the percentages of clinical remission were generally comparable among all subgroups, the percentage of complete mucosal healing was potentially influenced by the MMDAI and the history of previous medication by 5-ASA enemas or suppositories in the recent active phase. The percentage of complete mucosal healing in the subgroup of patients with MMDAI of 6–8 [32.3%, 10 of 31] was apparently lower than that in the subgroup of patients with MMDAI of 3–5 [64.0%, 16 of 25], suggesting that budesonide foam is more effective in inducing complete mucosal healing in patients with mild UC than in patients with moderate UC. The subgroup of patients with previous use of 5-ASA enema or suppository in the recent active phase was considered to contain patients with insufficient effects or intolerant of 5-ASA enemas or suppositories. Remarkably, 58.1% of patients achieved complete mucosal healing in the subgroup of patients without previous use of 5-ASA enema or suppository in the recent active phase. Furthermore, even in the subgroup of patients with a previous use of 5-ASA enema or suppository in the preent active phase, 32.0% of patients treated with BID budesonide foam achieved complete mucosal healing. This result suggests that BID budesonide foam was a good therapeutic option for UC patients with unacceptable or ineffective experience of 5-ASA enema or suppository use. The proximal extent of the colonic mucosal inflammation in UC patients varies among individuals, but it usually involves a lesion in the area from the sigmoid colon to the rectum because inflammation often extends distal to proximally in UC patients.^16,17,18^ Thus, complete mucosal healing of distal lesion is an important therapeutic target for a large majority of UC patients.

Our study was the first trial to evaluate the efficacy of budesonide foam to induce clinical remission in Japanese UC patients. On the basis of our results, both QD and BID dosages of budesonide foam were confirmed to be significantly effective compared with placebo in terms of clinical remission after 6 weeks of treatment. In a previous study in patients with active ulcerative proctitis or proctosigmoiditis, the clinical remission rates [defined as disease activity index ≤ 3] for QD 6-week treatment of budesonide 2-mg foam and budesonide 2-mg enema were 57% and 64%, respectively.^10^ In two recent randomiszed, placebo-controlled phase 3 studies in the USA, the efficacy and safety of budesonide 2-mg foam treatment BID for 2 weeks and then QD for 4 weeks were evaluated in patients with active, mild to moderate ulcerative proctitis or proctosigmoiditis. In these studies, the percentages of patients with clinical remission [defined as a rectal bleeding subscore of 0, endoscopic subscore ≤ 1, and the decrease of ≥ 0 in the stool frequency subscore from baseline] for budesonide 2-mg foam and placebo foam were 38.3% and 25.8%, respectively [study 1], and 44.0% and 22.4%, respectively [study 2].^11^ Although there are some differences in the definition of clinical remission between studies, the clinical remission rate in Japanese UC patients treated with budesonide foam for 6 weeks was considered generally consistent with previous reports. In the subgroup analysis, budesonide foam was confirmed to be effective in patients with a previous use of 5-ASA enemas or suppositories in the present active phase as well as patients with no previous use of them. Rectal corticosteroids are often used for patients with intolerant or insufficient response to 5-ASA enema or suppository.^19^ Thus, our results suggested the great benefit of budesonide foam for inducing clinical remission for these patients as well.

The assessment of safety and patient acceptance showed that budesonide foam is well tolerated in patients with QD administration and in those with BID administration. No serious adverse event was reported in any group. Although a decrease of the plasma cortisol and plasma corticotrophin levels was reported in patients in the budesonide foam groups, all recovered to normal levels after completing treatment. There was no evidence of any other increase of adverse event caused by glucocorticoids in either of the budesonide foam groups. The safety profile in this study was consistent with the worldwide experience of budesonide foam and oral budesonide formulation for UC.^11,20,21^ These results indicated that BID and QD budesonide foam were well tolerated.

Foam preparation is more convenient to administer and is better tolerated compared with a liquid enema. Because foam formulations can remain in contact with the mucosa for a long time, foam preparations are easier to maintain without leakage of the drug. Furthermore, the administration of foam preparation can be easily performed in the standing position, whereas liquid enema preparations must be administered with patients lying down. Actually, it has been reported that 84% of UC patients preferred foam preparations whereas enemas were only preferred by 6%, in a randomised study from Europe, demonstrating that foam preparation resolves problems related to liquid enema preparations.^10^ In this study, a direct comparison between budesonide foam and other enemas was not performed. However, the results of our questionnaires were generally consistent with those of patients using budesonide foam, in a previous report.^10^ Other enemas approved in Japan were commonly used by the QD administration. Hence, we were concerned that BID administration might be considered unacceptable by patients who were familiar with other enemas. Nevertheless, > 65% of patients answered none or slight to the questionnaire regarding difficulty with BID administration, regardless of their experience with other enemas. This result supports the conclusion that budesonide foam was generally accepted by patients even if they were required to receive it BID for 6 weeks.

In summary, our new results suggest that BID administration of budesonide foam effectively induced complete mucosal healing. BID administration of budesonide foam was not associated with any serious problems related to safety and application. BID budesonide foam has the possibility to be a therapeutic option for achieving complete mucosal healing in patients with mild to moderate UC.

## Funding

This work was supported by Ajinomoto Pharmaceuticals Co.

## Conflict of Interest

YS received lecture fees from Mitsubishi Tanabe Pharma Corporation, AbbVie Japan Co., and Zeria Pharmaceutical. KW received research grants from AbbVie, Mitsubishi Tanabe Pharma Corporation, Eisai, JIMRO, Asahi Kasei, Ajinomoto Pharmaceuticals, Kyowa Hakko Kirin, Kyorin Pharmaceutical, Astellas Pharma, UCB Japan, Takeda Pharmaceutical, Dainippon Sumitomo Pharma, Horii Pharmaceutical, fees for clinical contracts from Takeda Pharmaceutical, Janssen Pharmaceutical, and Pfizer Japan, and consulting fees from AbbVie, Mitsubishi Tanabe Pharma Corporation, Eisai, JIMRO, Asahi Kasei Medical, Ajinomoto Pharmaceuticals, Kyowa Hakko Kirin, Kyorin Pharmaceutical, Astellas Pharma, and UCB Japan. TH received research grants from AbbVie, Eizai, JIMRO, Otsuka Pharma, Mitsubishi Tanabe Pharma, and Zeria Pharmaceutical, and consulting fees from AbbVie, Astra Zeneca Pharmaceutical, Eizai, JIMRO, Otsuka Pharma, Mitsubishi Tanabe Pharma, Zeria Pharmaceutical, Kyorin Pharmaceutical, Pfizer Japan, and Janssen Pharmaceutical.

## Author Contributions

All authors were equally involved in: the concept and design of the study; the acquisition, analysis, and interpretation of data; drafting and revising the manuscript; and providing final approval of the version to be submitted.
